# Plain Talk on "Failures."

**Published:** 1892-08

**Authors:** E. D. Swain


AMERICAN JOURNAL

—OF—

DENTAL SCIENCE.

VOL. XXVI. BALTIMORE, AUGUST, 1892. NO. 4.

ARTICLE I.

### PLAIN TALK ON "FAILURES."

I do not care to discuss the question as to whether soft or cohesive gold should be used, hand or mallet pressure, all gold and no amalgam, beyond the statement that the dental practitioner who does not use all of them, as indicated, makes a deplorable failure and the practitioner who sees no good in a saved tooth, because its salvation was accomplished with a material other than gold, to my mind fails to comprehend his true mission in the profession he has chosen. I well remember cases in the earlier years of my own practice, whose ghosts haunt me now where failure of judgment, failure to use gutta-percha, oxy-chloride of zinc, or amalgam, cost my patient their natural organs and caused me the mortification of knowing that another failure was charged against me. Contour fillings of gold are good, and the theory that all teeth filled should be restored to their original pristine beauty in form, etc., *sounds* good certainly, especially as it is sometimes elaborately described to us by gentlemen competent to talk upon such subjects. Still every one of you have seen the most outrageous failures from this source simply because the operator was proud of the fact that he could make a beautiful filling in a very frail tooth; deplorable failure in judgment as well as the service rendered.

Within a year I met a man and brother dentist who has been in practice counting his college experience not more than five years, who after some general conversation upon dental operations said: "I have seen some of your operations recently, made several years ago, and could not help criticising them." I asked in what respect; the reply was "you cut away too freely." I asked if the operations were saving the teeth? He replied, "Yes, I found only one which required a slight repair at the cervical margin." He then volunteered the information that in those bicuspids and molars where the dentine had been destroyed by disease, leaving the enamel standing, he tried to preserve that rather than cut it away, thereby exposing so much gold. My word for it, if this man continues in practice he will have some failures which will annoy him and cause his patients to speak after the manner of men, as forbidden in the fifth commandment.

Prof. Black has recently, in a series of articles in the *Cosmos*, given us advice upon the preparation of enamel edges, which if followed would save us many failures in this direction.

Another *growing* cause of failure, arises from the indiscriminate use of the dental engine and burs in the preparations of cavities for filling; I have seen so many instances where the cavity was the shape of a round bur, the enamel edges so thin that the most delicate manipulation with the finest instruments could not prevent their fracture, also in many localities it would be impossible to pack the gold into the undercuts so made. I have always feared the use of the bur in deep cavities, or in those where it was dangerous to cut to any considerable depth, the hole being filled with the fine cuttings of the dentine, making it next to impossible to know what one is doing.

Recently two sisters applied to me for advice regarding their incisor teeth, which were considerably discolored, the usual tests convinced me that the pulps were dead. I removed some of the fillings verifying my diagnosis in this direction and finding the cavities formed as I have mentioned above, when burs were used. I commenced a course of questioning, which proved to me that these cavities were wholly prepared with burs, and that in each instance an exposure of the pulp was the result, consequently abscesses, discoloration and disfigurement.

A prominent dentist and careful operator in my office recently remarked "that some operators seemed to feel that they knew just where to look for a pulp canal and seemed to think that when necessary to open them for treatment, they could with a very fine drill and the engine, go through the crown and hit them every time;" his experience had been, however, that they as often went through the side of the root, through between the roots at the bifurcation, or at some other point, endangering the chances of saving the tooth. This is even a greater failure than those already mentioned, arising from too free use of the dental engine. Another dentist present a teacher, suggested that students should not be allowed the use of the engine during the first two years of their pupilage. Another, a Professor in one of our Dental Schools, not willing to go quite so far, was of the opinion that more attention should be given this subject in the education of dental students. I do not condemn the use of the engine, or burs; instead, I find them very valuable aids, but if we would avoid such failures as mentioned, we should use great care and discrimination when we do use them.

Another source of many failures I believe arises from the use of deeply serrated gold packing instruments. It can be readily understood, that where comparatively large pellets are used, the long, sharp points pass entirely through, and not only fail to pack the gold against the walls of the cavity, but even pulverize them, leaving under the gold a layer of chips of dentine, thereby preventing the possibility of a moisture proof filling; and furthermore I have come to believe that much of the discoloration seen beneath thin walls, especially in incisor teeth, is due to the failure to remove all the dust produced by preparing the cavity.

Why do we speak of recurring decay about a filling of years standing as a failure, especially if that tooth has been filled with gold ? Has this material properties in its pure metallic state which will prevent caries of the teeth ? It is not antiseptic, and only answers the purpose best because it is soft and can be coaxed to remain in place when once fixed, if properly done; excluding moisture, particles of food and decomposible secretions which are always present in the mouth, the same conditions exist, the same tooth is composed of the same materials, weakened in its powers to resist diseased conditions now because of the disease infecting it years ago; to convey the idea to our patient that a tooth once filled with gold, is in the future safe from further disease, is one of our greatest failures as professional men. Did you ever hear of a physician assuring his patient that when he had once mastered the present attack, they would be exempt from similar ones in the future ? That a bone once healed would never break again ?

Is it not true that one has to grow old in practice, before he has the courage to say to his patron, "little of the work I do for you can be pronounced other than temporary, and in proportion as your teeth are now liable to caries will these operations require to be repeated;" here we fail in courage.—SWAIN (E. D.), *Dental Review.*

